# Molecular mechanisms of hepatic lipid accumulation in non-alcoholic fatty liver disease

**DOI:** 10.1007/s00018-018-2860-6

**Published:** 2018-06-23

**Authors:** David Højland Ipsen, Jens Lykkesfeldt, Pernille Tveden-Nyborg

**Affiliations:** Department of Veterinary and Animal Sciences, Faculty of Health and Medical Sciences, University of Copenhagen, Ridebanevej 9, 1870 Frederiksberg C, Denmark

**Keywords:** Lipid metabolism, Animal models, Pharmacotherapy

## Abstract

Non-alcoholic fatty liver disease (NAFLD) is currently the world’s most common liver disease, estimated to affect up to one-fourth of the population. Hallmarked by hepatic steatosis, NAFLD is associated with a multitude of detrimental effects and increased mortality. This narrative review investigates the molecular mechanisms of hepatic steatosis in NAFLD, focusing on the four major pathways contributing to lipid homeostasis in the liver. Hepatic steatosis is a consequence of lipid acquisition exceeding lipid disposal, i.e., the uptake of fatty acids and de novo lipogenesis surpassing fatty acid oxidation and export. In NAFLD, hepatic uptake and de novo lipogenesis are increased, while a compensatory enhancement of fatty acid oxidation is insufficient in normalizing lipid levels and may even promote cellular damage and disease progression by inducing oxidative stress, especially with compromised mitochondrial function and increased oxidation in peroxisomes and cytochromes. While lipid export initially increases, it plateaus and may even decrease with disease progression, sustaining the accumulation of lipids. Fueled by lipo-apoptosis, hepatic steatosis leads to systemic metabolic disarray that adversely affects multiple organs, placing abnormal lipid metabolism associated with NAFLD in close relation to many of the current life-style-related diseases.

## Introduction

Affecting 25% of the adult population, non-alcoholic fatty liver disease (NAFLD) is currently the most common liver disease in the world [1]. Regional prevalence rates are currently highest in the Middle East (32%) and South America (30%) and lowest in Africa (13%), but prevalence rates are even higher in specific subpopulations such as severely obese (90%) and patients with type 2 diabetes (76%) [1]. Furthermore, NAFLD in lean individuals is far from uncommon with prevalence rates around 16% [2, 3]. NAFLD is associated with increased mortality, particularly due to cardiovascular disease, hepatocellular carcinoma, and liver-related events [4]. The escalating prevalence, particularly during the last decades, has made NAFLD the second most common cause of liver transplantation in the United States [5]. The hallmark of NAFLD is hepatic steatosis, but the disease also encompasses non-alcoholic steatohepatitis (NASH) characterized by hepatic inflammation, hepatocyte damage, and fibrosis, highlighting the potentially progressive nature of the disease. The stage of hepatic fibrosis predicts both overall and liver-related mortality and is the strongest predictor of long-term clinical outcomes, with advanced fibrosis (stages 3 and 4) conveying the highest risk of mortality [6]. However, progression to fibrosis also occurs in patients with steatosis alone [7], although rates of progression and overall mortality rates are increased in NASH [1, 8]. In addition, metabolic dysfunctions, such as insulin resistance, dyslipidemia, and cardiovascular disease are all associated with hepatic steatosis, and seem to be more related to hepatic fat accumulation and NAFLD than obesity status per se [2, 9, 10].

**Fig. 1:**
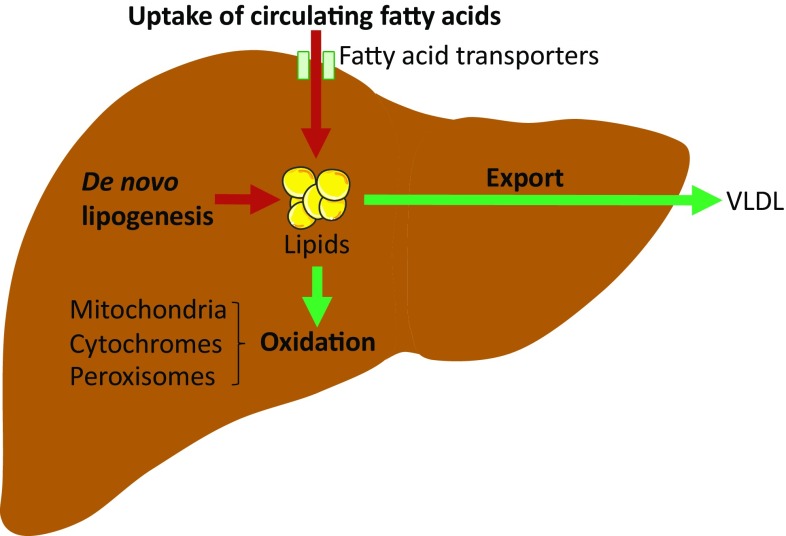
Hepatic lipid acquisition and disposal. Intrahepatic lipid levels are governed by the balance between lipid acquisition and disposal constituting the four major pathways of hepatic lipid homeostasis. The liver acquires lipids through the uptake of circulating fatty acids and via de novo lipogenesis. Conversely, lipids may be disposed of through oxidation (in the mitochondria, peroxisomes and cytochromes) and through export as very low density lipoprotein (VLDL) particles. Consequently, lipid accumulation is the result of lipid acquisition pathways exceeding disposal pathways

**Fig. 2:**
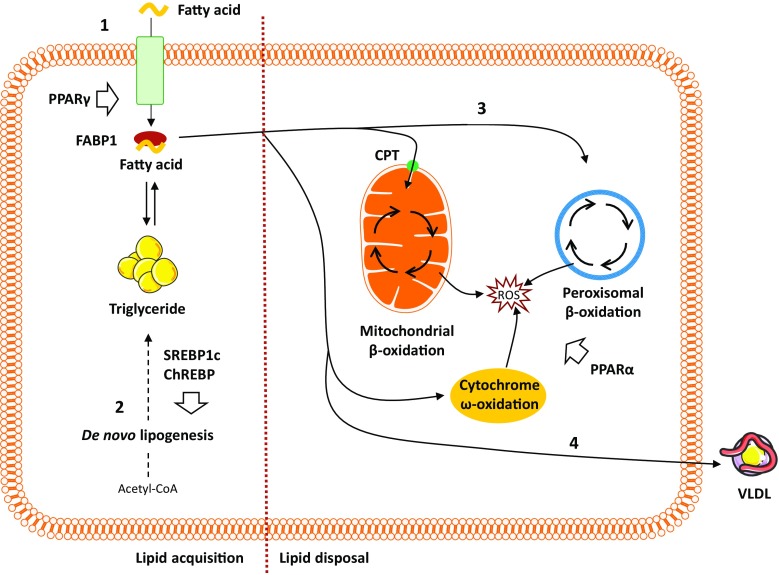
Overview of hepatic lipid metabolism. (1) Uptake of circulating lipids are facilitated by specific fatty acid transporters located in the hepatocyte plasma membrane and is regulated by PPARγ. FABP1 facilitates the transport of hydrophobic fatty acids to the different cellular compartments within the cytoplasm. (2) De novo lipogenesis converts acetyl-CoA (originating from excess carbohydrates) to new fatty acids, which subsequently can be esterified and stored as triglycerides. Regulation of de novo lipogenesis is complex, but broadly controlled by two key transcription factors: SREBP1c and ChREBP. (3) Fatty acid oxidation is controlled by PPARα and reduces intrahepatic fat levels by utilizing lipids as an energy source. While the process primarily occurs in the mitochondria, lipid overload and/or compromised mitochondrial function forces a higher degree of fatty acid oxidation to take place in the peroxisomes and cytochromes, thereby, generating ROS. (4) The liver can export lipids by packaging them into water-soluble VLDL-particles, which may then be utilized or stored in other tissues. *ChREBP* carbohydrate regulatory element binding protein, *CPT* carnitine palmitoyltransferase, *FABP* fatty acid binding protein, *PPAR* peroxisome proliferator-activated receptor, *ROS* reactive oxygen species, *SREBP1c* sterol regulatory element binding protein 1c, *VLDL* very low density lipoprotein

The liver constitutes an essential organ in lipid metabolism. As a central regulator of lipid homeostasis, the liver is responsible for orchestrating the synthesis of new fatty acids, their export and subsequent redistribution to other tissues, as well as their utilization as energy substrates [11] (Fig. 1). These processes are closely regulated by complex interactions between hormones, nuclear receptors, and transcription factors, keeping hepatic lipid homeostasis under tight control [12]. The disruption of one or more of these pathways may precipitate the retention of fat within the liver and the subsequent development of NAFLD. Hepatic fat accumulation results from an imbalance between lipid acquisition and lipid disposal, which are regulated through four major pathways: uptake of circulating lipids, de novo lipogenesis (DNL), fatty acid oxidation (FAO), and export of lipids in very low-density lipoproteins (VLDL) (Fig. 2). However, the molecular mechanisms underlying the pathological aggregation of fat within the liver are not fully elucidated. This review explores current insights to these four pathways and the molecular mechanisms regulating hepatic lipid homeostasis in NAFLD, discussing processes that may be instrumental in the development and progression of hepatic steatosis.

## Hepatic lipid uptake

**Fig. 3:**
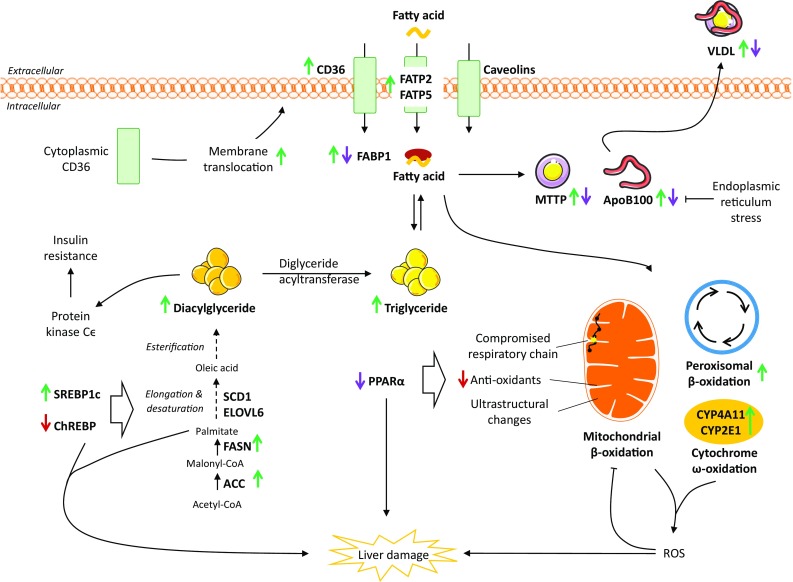
Effects on hepatic lipid metabolism in NAFLD. While the role of hepatic caveolins is still unclear, CD36, FATP2 and -5 mediates increased uptake of circulating lipids in NAFLD. Initially, FABP1 is increased, but levels may decline with disease progression, potentially limiting the mobility of fatty acids and sustaining steatosis. Enhanced SREBP1c-mediated de novo lipogenesis is a key feature of NAFLD contributing significantly to the accumulation of lipids. At the same time, ChREBP which could be hepatoprotective, appears to be downregulated in NAFLD. Although data relating to the regulation of fatty acid oxidation are conflicting, mitochondrial dysfunction is an important feature of NAFLD resulting in increased generation of ROS and utilization of cytochrome- and peroxisome-mediated oxidation. This further promotes oxidative stress, in turn inducing damage to the mitochondrial membranes, compromising cellular respiration and metabolism, and impairing liver function by direct and indirect cellular damage. Lastly, lipid export increases with hepatic triglyceride levels. However, in the setting of NASH, levels of MTTP and apoB100 may be decreased, hereby, limiting VLDL export and instead facilitating lipid accumulation. The net result is an escalating vicious circle, driven by chronic dyslipidemia and hepatic lipid overload, leading to detrimental consequences for liver metabolism and function and ultimately promoting irreversible liver damage. Green arrow: increased expression. Red arrow: decreased expression. Purple arrow: expression different between steatosis and NASH. *ACC* acetyl-CoA carboxylase, *ApoB100* apolipoprotein B100, *CD36* cluster of differentiation 36, *ChREBP* carbohydrate regulatory element binding protein, *ELOVL* elongation of very long chain fatty acid, *FABP* fatty acid binding protein, *FASN* fatty acid synthase, *FATP* fatty acid transport protein, *MTTP* microsomal triglyceride transfer protein, *PPAR* peroxisome proliferator-activated receptor, *ROS* reactive oxygen species, *SCD1* stearoyl-CoA desaturase 1, *SREBP1c* sterol regulatory element binding protein 1c, *VLDL* very low density lipoprotein

The uptake of circulating fatty acids by the liver is largely dependent on fatty acid transporters, while passive diffusion contributes less [13]. The transport is predominately mediated by fatty acid transport proteins (FATP), cluster of differentiation 36 (CD36), and caveolins located in the hepatocyte plasma membrane [14] (Fig. 3). Of the six mammalian FATP isoforms, FATP2 and FATP5 are found primarily in the liver [14]. Knockdown of FATP2 in mice decreases uptake of fatty acids and ameliorates hepatic steatosis induced by a high fat diet [15]. Likewise, knockout or knockdown of FATP5 in mice reduces hepatocyte fatty acid uptake, hepatic triglyceride content, and reverses steatosis [16, 17] indicating a role of FATP-mediated lipid uptake as a facilitator of hepatic steatosis. FATP2 and 5 gene expression was increased in adolescents with NASH (*n* = 27) compared to normal controls (*n* = 6) [18]. In contrast, a small study found no difference in hepatic FATP5 gene expression between individuals with (*n* = 16) and without (*n* = 8) hepatic steatosis [19]. FATP5 promotor polymorphism (rs56225452), representing a putative gain-of-function mutation in the FATP5 promotor, correlated with BMI-dependent hepatic steatosis in males with biopsy proven NAFLD (*n* = 103) [20], suggesting that genetic variation may underlie part of the contribution of FATP5 in NAFLD. However, additional studies are required to extend our present understanding of the role of FATP in clinical NAFLD.

The fatty acid translocase protein, CD36, facilitates the transport of long-chain fatty acids and is regulated by peroxisome proliferator-activated receptor (PPAR) γ, pregnane X receptor, and liver X receptor [21]. Mice fed a high fat diet develop hepatic steatosis alongside increased mRNA and protein expression of CD36 [22, 23]. Adenovirus-mediated overexpression of CD36 enhances hepatic fatty acid uptake and fat accumulation [22], while liver-specific knockout of CD36 decreases hepatic lipid levels in both genetic and diet-induced steatosis [23]. This suggests a causal role of CD36 in steatosis supported by abnormally increased CD36 levels in NAFLD patients: a small study reported increased CD36 mRNA levels in obese subjects with high amounts of intracellular fat (66.0 ± 6.8%, *n* = 5), compared to subjects with low liver fat content (6.4 ± 2.7%, *n* = 5) [24]. In adolescents and adults of both genders, diagnosed with NASH or hepatic steatosis, CD36 gene and protein expression were increased compared to healthy controls; however, CD36 levels did not differ between patients with steatosis and NASH [18, 25]. In contrast, hepatic levels of CD36 were found to be similar in morbidly obese women with hepatic steatosis compared to morbidly obese controls with normal livers [26]. However, the conflicting findings of expression patterns may not adequately illustrate a functional consequence of the role of CD36. Immunohistochemistry of liver sections showed CD36 as located in the hepatocyte plasma membrane in patients with steatosis and NASH, compared to a weak CD36 expression confined to the cytoplasm of hepatocytes in normal livers, suggesting that the translocation of CD36 protein from cytoplasm to membrane may be a triggering event in NAFLD progression [25].

The caveolins comprise a family of three membrane proteins contributing to lipid trafficking and formation of lipid droplets [14]. Caveolin 1 was increased in the liver of mice with NAFLD, and located mainly in the centrilobular zone 3, where the steatosis was most severe [27]. Similarly, zone 3 predominant hepatic steatosis is reported in adult NAFLD patients [28]. Whole-body caveolin 1 knockout (c*av1*^−*/*−^) reduced hepatic steatosis in high fat fed mice in response to 24 h of fasting, whereas liver-specific caveolin 1 knockout did not affect hepatic fat content [29]. Reduced hepatic steatosis in fasted c*av1*^−*/*−^ mice was suggested to be secondary to compromised metabolic function in the adipose tissue, resulting in reduced hepatic DNL (and possibly increased FAO) [29]. In contrast, decreased caveolin 1 expression has been reported in the livers of high fat fed mice with NAFLD [30]. Furthermore, caveolin 1 knockout augmented steatosis both in vitro and in vivo by enhancing the expression of genes involved in DNL [30], contradicting earlier studies, and suggesting a protective effect of caveolin 1 in NAFLD [30]. Differential observations suggest that the role of caveolin 1 in hepatic lipid accumulation may differ depending on how the steatosis is induced, e.g., via high fat diet or fasting. However, long-term fasting is typically not the primary cause of NAFLD, and results from such models should consequently be translated with caution.

Following uptake, hydrophobic fatty acids do not diffuse freely in the cytosol and must instead be shuttled between different organelles by specific fatty acid binding proteins (FABP) of which FABP1, also known as liver FABP, is the predominant isoform in the liver [13]. FABP1 facilitates the transportation, storage, and utilization of fatty acids and their acyl-CoA derivatives and may exert a protective effect against lipotoxicity by binding otherwise cytotoxic free fatty acids and facilitating their oxidation or incorporation into triglycerides [31]. FABP1 also affects the expression of PPARα and PPARγ by mediating the transport of PPAR ligands to the nucleus of hepatocytes, and intracellular FABP1 concentrations are correlated with PPARα and PPARγ activities [32]. Hepatic triglycerides and lipid disposal pathways (fatty acid export and oxidation) are decreased following FABP1 ablation in fasted mice, suggesting that reduced levels of liver triglycerides are linked to a reduced hepatic lipid uptake, at least in the fasted state in which lipid flux to the liver is increased [33, 34]. In patients with NAFLD, hepatic FABP1, FABP4, and FABP5 mRNA levels were increased compared to non-NAFLD controls and FABP4 and 5 correlated with the percentage of liver fat [19, 35]. Thus, enhanced intracellular trafficking of fatty acids in the lipid laden liver of NAFLD patients may be shunting harmful fatty acids to storage, thereby promoting steatosis. FABP levels may vary according to disease severity as FABP1 protein levels were overexpressed in obese patients with steatosis (*n* = 10) compared to obese controls (*n* = 10), but decreased in NASH patients with mild fibrosis (*n* = 10) and declining further in NASH with advanced fibrosis (*n* = 10) [36]. Thus, increased FABP1 in the earlier stages of NAFLD may enhance lipid flux as a compensatory mechanism to limit lipotoxicity. As the disease progresses, diminishing levels of FABP1 potentially leads to increased levels of lipids, with ensuing lipotoxicity promoting disease progression by damaging essential organelles and cells in the liver (Fig. 3).

## De novo lipogenesis

In short, DNL enables the liver to synthesize new fatty acids from acetyl-CoA. Initially, acetyl-CoA is converted to malonyl-CoA by acetyl-CoA carboxylase (ACC) and malonyl-CoA is then converted to palmitate by fatty acid synthase (FASN). New fatty acid may then undergo a range of desaturation, elongation, and esterification steps before ultimately being stored as triglycerides or exported as VLDL particles (Fig. 3). Thus, increased DNL can cause hepatic steatosis and/or hypertriglyceridemia, but may also induce steatohepatitis, as saturated fatty acids, such as palmitate, can cause inflammation and apoptosis [37]. Studies using stable isotope tracers suggest that an important characteristic of NAFLD patients is abnormally elevated DNL regardless of fasting. Accordingly, a small study reported increased DNL in NAFLD patients (*n* = 5) compared to controls (*n* = 6) [38]. These findings are supported by enhanced DNL in overweight/obese subjects with high liver fat (18.3 ± 3.6%, *n* = 13) compared to those with lower liver fat (3.1 ± 2.7%, *n* = 11) while being matched for adiposity and circulating lipids [39]. In addition, DNL was independently associated with intrahepatic triglyceride levels [39], and inadequate suppression of DNL during fasting could be a key feature in NAFLD patients [38, 39]. The importance of DNL in NAFLD is further supported by studies, showing that in obese patients with NAFLD, approximately 26% of hepatic triglycerides were derived from DNL and that these patients were unable to regulate DNL when transitioning from fasting to fed state [40]. While limited, the available clinical data collectively indicate that failure to regulate DNL is a central feature of liver lipid accumulation in NAFLD patients.

The transcriptional regulation of DNL is mainly orchestrated by two key transcription factors: sterol regulatory element-binding protein 1c (SREBP1c), which is activated by insulin and liver X receptor α, and carbohydrate regulatory element-binding protein (ChREBP), which is activated by carbohydrates [41–43] (Fig. 2). SREBP1c expression is enhanced in patients with NAFLD, and in agreement with its lipogenic role, hepatic triglyceride levels are higher in transgenic mice overexpressing SREBP1c [44, 45], while SREBP1c knockout mice display decreased expression of lipogenic enzymes [46]. SREBP1c ablation also promotes a compensatory upregulation of SREBP2, leading to increased hepatic cholesterol synthesis and cholesterol accumulation, linking DNL to cholesterol metabolism [46]. While the phenotypic insulin resistance in NAFLD would be expected to counteract insulin-mediated SREBP1c activation, a state of selective insulin resistance ensures that insulin retains its ability to promote DNL through SREBP1c while being unable to suppress gluconeogenesis [41]. This helps may explain the observed elevated rates of hepatic DNL under insulin resistant conditions. In addition, SREBP1c indirectly contributes to the development of hepatic insulin resistance, since enhanced lipogenesis and subsequent accumulation of harmful lipid species, such as diacylglycerides, may interfere with insulin signaling (as discussed below). ChREBP mediates carbohydrate, but not fat-induced DNL as high fat diets do not activate ChREBP and may even decrease ChREBP activity [43, 47]. In mice, knockout of ChREBP has been shown to reduce hepatic fatty acid synthesis by 65% compared to wild-type controls, but also to promote insulin resistance, delayed glucose clearance, and severe intolerance to simple carbohydrates, such as sucrose and fructose (resulting in death in the majority of animals) due to an inability to shunt fructose into glycolytic pathways [48]. This emphasizes the essential role of ChREBP in both lipid and glucose metabolisms and suggests that ChREBP is required for a normal lipogenic response following the ingestion of carbohydrates [48]. In *ob/ob* mice, silencing ChREBP reduces hepatic triglyceride content specifically through inhibition of glucose-induced lipogenesis [49]. Likewise, ChREBP knockout protected against fructose-induced steatosis in mice, but substantially increased histological liver damage as a result of enhanced cholesterol synthesis and subsequent cytotoxicity [50]. By limiting levels of cytotoxic-free cholesterol and the subsequent liver injury, ChREBP may confer a hepatoprotective effect [50]. Increased ChREBP levels in NAFLD could then constitute a potential defense mechanism shielding the liver from further injury and progression towards NASH. Supporting this concept, lipogenesis has been reported to be dissociated from NASH progression, i.e., elevated DNL may induce steatosis, but may be protective in relation to disease progression [51]. High ChREBP expression was found in biopsies from NASH patients, but levels declined in patients with severe insulin resistance, indicating that ChREBP may segregate hepatic steatosis from insulin resistance [52]. In high fat fed mice, adenovirus-mediated ChREBP overexpression resulted in hepatic steatosis and increased DNL. However, insulin sensitivity and glucose tolerance were maintained, likely owing to an increased conversion of saturated fatty acids (known to cause insulin resistance) to monounsaturated fatty acids mediated by stearoyl-CoA desaturase-1 (SCD1) [52]. ChREBP was down-regulated in patients with NAFLD (*n* = 22) compared to healthy controls (*n* = 10), and instead, SREBP1c was reported to be one of the predominant regulators of DNL in NAFLD, upregulating genes coding for ACC1 and FAS [53].

In response to elevated SREBP1c, the expression of downstream targets ACC and FASN was increased in both patients and animal models with NAFLD [18, 44, 45, 53–57]. Liver-specific knockout of ACC1 decreased hepatic lipid accumulation in mice and DNL in hepatocytes [58]. However, knockout mice were not protected from hepatic steatosis induced by a high fat diet, potentially due to decreased fatty acid oxidation caused by a compensatory increase of ACC2, which in turn inhibited mitochondrial β-oxidation [58]. Accordingly, inhibition of both ACC1 and ACC2 was required to improve hepatic steatosis in mice [59], implying that both isoforms are important in NAFLD. Paradoxically, liver-specific FASN knockout promoted hepatic steatosis in mice on a zero-fat diet [60], in which steatosis developed alongside defective PPARα signaling and with a phenotype that could be corrected by dietary fat or a PPARα agonist [60]. The study identified ‘new’ lipids, formed either through DNL or originating from the diet, as potential PPARα ligands contributing to the regulation of hepatic lipid homeostasis [60]. As mentioned, conversion of saturated fatty acids to mono-unsaturated fatty acids by SCD1 may be protective in NAFLD [52]. In agreement, incubation of hepatocytes with saturated fatty acids decreased cell viability, while incubation with mono-unsaturated fatty acids enhanced lipid accumulation without affecting cellular viability [61]. Despite preventing steatosis, SCD1 knockout exacerbated hepatic fibrosis and cellular apoptosis in mice with NASH induced by a methionine and choline deficient diet [61]. The end-result of SCD1 inhibition may, therefore, be an aggravation of NASH due to intracellular accumulation of cytotoxic saturated fatty acids [51, 62], placing the partitioning of saturated fatty acids to mono-unsaturated fatty acids as a protective factor in delaying NAFLD progression. In view of the considerable crosstalk between the molecular pathways in hepatic lipid homeostasis, inhibition of DNL is not a trivial task, even though it may constitute an attractive therapeutic target.

Ectopic lipid deposition directly promotes insulin resistance, which is a common feature of patients with NAFLD-associated diseases [63, 64]. Insulin sensitivity in liver, muscles, and adipose tissue was reduced in subjects with high amounts of hepatic lipids (25.3 ± 3.5%, *n* = 10) compared to individuals with normal levels of hepatic lipids (3.6 ± 0.5%, *n* = 10) matched for visceral adipose tissue volume [65]. Furthermore, hepatic insulin sensitivity was only compromised in obese individuals (*n* = 20) when NAFLD was present [66]. As not all patients with fatty livers develop NASH, some individuals must possess protective mechanisms to shield them from lipotoxicity, e.g., lipid desaturation and inhibition of lipid-induced inflammation [67]. In the promotion of insulin resistance, diacylglycerides have emerged as potential mediators. Diacylglycerides are precursors of triglycerides, and hepatic accumulation of diacylglycerides has been associated with hepatic insulin resistance through the induction of protein kinase Cε [68]. Transgenic mice overexpressing diglyceride acyltransferase 2—catalyzing the conversion of diglycerides to triglycerides—increased hepatic triglyceride content fivefold without affecting insulin sensitivity [69]. Likewise, antisense oligonucleotides against protein kinase Cε protected rats from diet-induced hepatic insulin resistance [70]. Conversely, inhibition of diglyceride acyltransferase 2 with antisense oligonucleotides in *db/db* mice fed a methionine and choline-deficient diet-reduced hepatic steatosis, but augmented hepatic inflammation, fibrosis, and apoptosis [71]. Based on magnetic resonance spectroscopy, livers of obese subjects were classified with no steatosis (< 5.56%, *n* = 52), mild steatosis (5.56–15%, *n* = 41), or severe steatosis (> 15%, *n* = 40), revealing that the presence of NAFLD, but not the amount of hepatic triglycerides, was associated with hepatic insulin resistance [72]. When investigating liver biopsies from a subset of the subjects (*n* = 27), only cytoplasmic diacylglyceride levels, and not total or membrane-associated diacylglycerides, predicted hepatic insulin resistance [72]. Enhanced membrane translocation of protein kinase Cϵ (i.e., activation) provides a potential mechanism for diacylglyceride-induced insulin resistance, suggesting hepatic diacylglyceride to be a relevant predictor of insulin resistance in NAFLD [72, 73]. In patients with steatosis (*n* = 9) or NASH (*n* = 9), hepatic diacylglyceride levels were equally increased compared to controls (*n* = 9) [74]. In addition, diglyceride acyltransferases 1 and 2 were not differentially expressed between patients with steatosis (*n* = 51) and NASH (*n* = 53) [75]. It appears that once steatosis is established, further NAFLD/NASH progression does not promote additional alterations in diacylglyceride-linked lipid metabolism, rendering diacylglycerides to exert an adverse effect already during the early stages of NAFLD development. The ongoing accumulation of hepatic triglyceride may represent a compensatory measure implemented to convey some degree of protection against more harmful lipid species. Though possibly an appealing thought, steatosis should not be interpreted as being beneficial, since chronic hepatic steatosis is associated with several serious conditions, including dyslipidemia and hypertension, imposing considerable negative effects on the quality of life as well as increasing mortality in afflicted patients [76].

Collectively, lipid accumulation in NAFLD is supported by enhanced lipogenesis, denoting DNL as a potential therapeutic target. However, blocking specific enzymes related to DNL may, in some cases, exacerbate NASH and the accompanying metabolic deterioration by promoting accumulation of cytotoxic lipid species, indicating the importance of the composition of the fatty acid pool in the liver (Fig. 3).

## Oxidation of fatty acids

Oxidation of fatty acids is controlled by PPARα and occurs mainly in the mitochondria, providing a source of energy to generate ATP especially when circulating glucose concentrations are low [14, 77–81]. In mammalian cells, the mitochondria, peroxisomes, and cytochromes mediate FAO [78, 82]. Entry of fatty acids into the mitochondria relies on carnitine palmitoyltransferase 1 (CPT1) situated in the outer mitochondrial membrane [80], but as the mitochondria lack the ability to oxidize very long chain fatty acids [79], these are preferably metabolized via peroxisomal β-oxidation. In case of lipid overload—such as in NAFLD—ω-oxidation in the cytochromes also contributes [78]. However, these processes generate considerable amounts of reactive oxygen species (ROS), oxidative stress, and toxic dicarboxylic acids, potentially promoting inflammation and disease progression [78] (Fig. 3).

Activation of PPARα induces the transcription of a range of genes related to FAO in the mitochondria, peroxisomes, and cytochromes, thereby reducing hepatic lipid levels [77, 80, 81, 83]. Knockout of PPARα in *ob/ob* mice results in hepatic steatosis, supporting a role of PPARα in the regulation of hepatic lipid metabolism [84]. A 24 h fasting period of WT or *ob/ob* mice, upregulated PPARα and PPARα-target genes related to mitochondrial (medium- and long-chain acyl-CoA dehydrogenases), peroxisomal (acyl-CoA oxidase (ACOX) 1 and enoyl-CoA hydratase), and cytochrome-mediated (CYP4A1 and CYP4A3) FAO [85]. This response was less pronounced in PPARα knockout animals and coincided with hepatic steatosis, again emphasizing the critical role of PPARα in promoting FAO and preventing hepatic lipid accumulation [85]. In humans, hepatic PPARα levels did not differ between patients with steatosis (*n* = 16) and healthy controls (*n* = 8) [19]. However, PPARα was downregulated in patients with NASH compared to patients with steatosis and healthy controls [75, 86], and the expression of PPARα decreased with increasing NAFLD activity score and fibrosis stage [86]. In addition, a longitudinal analysis after a 1-year follow-up associated increased PPARα with histological improvements in NASH [86]. Decreased PPARα in NASH also enhanced the DNA-binding capacity of c-Jun N-terminal kinase 1 (JNK1) and nuclear factor kappa-light-chain enhancer of activated B cells (NF-κB) leading to increased hepatic inflammation [87]. Thus, PPARα expression may be related to several aspects of NASH progression, modulating not only lipid homeostasis, but inflammation as well.

The expected consequence of hepatic lipid accumulation would be increased FAO. However, studies of FAO in patients with steatosis or NASH are conflicting, reporting enhanced [88–92], unchanged [93], or decreased FAO [94]. The broad range of hepatic states covered by the term NAFLD makes it difficult to compare studies directly, and oxidation of fatty acids may differ based on the severity of the disease. Furthermore, FAO capacities may vary inter-individually rendering some subjects more susceptible to NAFLD. Indeed, rats selectively bred for low intrinsic aerobic capacity display decreased mitochondrial FAO and were predisposed to diet-induced hepatic steatosis [95]. Expression of genes related to mitochondrial and peroxisomal β-oxidation was higher in patients with more severe steatosis (*n* = 11) compared to patients with less severe steatosis (*n* = 21) or non-steatotic controls (*n* = 16) [96]. β-Oxidation, measured indirectly as plasma β-hydroxybutyrate levels, was higher in patients with NASH (*n* = 6) compared to steatosis (*n* = 6) or normal controls (*n* = 6) [90]. Increased FAO may be an adaptive response in patients with NAFLD attempting to reduce the lipid overload and lipotoxicity, but also produces ROS and excessive FAO may overwhelm the capacity of the anti-oxidant defense system and induce oxidative stress. Accordingly, hepatic oxidative stress and changes in mitochondrial ultrastructure were increased alongside FAO in patients with NASH [90]. Glutathione, glutathione peroxidase, and superoxide dismutase were decreased in liver biopsies from NAFLD patients and in mitochondria from animal models of NAFLD [82, 87], closing the loop on a vicious cycle in which the diminishing capacity of the antioxidant defense system is continuously being depleted by rising ROS levels. In NASH patients (*n* = 10), oxidative DNA damage was significantly increased compared to healthy controls (*n* = 16) despite similar rates of FAO following an intravenous infusion of lipids, suggesting increased susceptibility to oxidative stress in these patients [89].

Lipid oxidation and oxidative damage to mitochondrial DNA further diminish mitochondrial function, establishing a self-perpetuating vicious cycle to exacerbate mitochondrial dysfunction and oxidative stress [80]. Decreased activity of the mitochondrial respiratory chain was reported in overweight/obese patients with NASH compared to controls [97, 98], and alterations in mitochondrial ultrastructure have been observed prior to NAFLD development in the Otsuka Long–Evans Tokushima Fatty rat [99] as well as in patients with steatosis preceding progression to NASH [100]. The decrease in mitochondrial function may result in the utilization of alternative FAO pathways. Mice heterozygous for mitochondrial trifunctional protein have compromised mitochondrial β-oxidation and develop hepatic steatosis alongside a compensatory upregulation of CYP2E1-facilitated FAO and oxidative stress [101, 102]. In NASH patients, hepatic CYP2E1 activity was increased and expression specifically localized to steatotic areas compared to patients with steatosis and healthy controls [103–105]. CYP2E1 activity correlated with disease severity suggesting an involvement of CYP2E1 in FAO particularly during later disease stages, i.e., NASH [104]. In contrast, other studies have reported no difference in CYP2E1 expression between patients with NASH (*n* = 30) and those with only steatosis (*n* = 10) [106]. Thus, enhanced CYP2E1 did not differentiate bland steatosis from NASH, but could still play a role in disease progression. In agreement with enhanced cytochrome-mediated FAO, increased CYP4A11—another key fatty acid metabolizing enzyme located in the cytochromes—has been reported in patients with NAFLD [45, 107]. Increased FAO in cytochromes may then be an important event in steatosis and NASH, with the excessive amount of ROS produced by the CYP enzymes exacerbating hepatic oxidative stress and consequently worsening liver damage.

The last of the three organelles important to fatty acid metabolism and hepatic lipid homeostasis is the peroxisomes. Targeting this system, either by hepatocyte-specific knockout of peroxisomes or by deficiency in ACOX (which catalyzes the initial step in peroxisomal FAO), results in hepatic lipid accumulation and fibrosis, oxidative stress, and inflammation, emphasizing the role of peroximal FAO in NAFLD and NASH [108]. A massive upregulation of PPARα was observed during ageing in ACOX deficient mice, suggesting ACOX substrates as endogenous activators of PPARα [109]. ACOX and branch-chained acyl-CoA oxidase (another peroxisomal enzyme involved in FAO) mRNA levels were higher in patients with NAFLD compared to controls, indicating that peroxisomal FAO upregulation may be a compensatory response aiming to resolve the progressing steatosis in NAFLD [45, 107]. However, similar to ω-oxidation in the cytochromes, the peroxisomes generate ROS as they oxidize fatty acids, and likewise, the peroxisomes may induce oxidative stress and promote disease progression [83].

In summary, the current data on FAO in NAFLD are conflicting, but even in studies suggesting enhanced FAO, augmented oxidation of fatty acids appear inadequate in clearing the liver of lipids. FAO in dysfunctional mitochondria—a characteristic of NAFLD—produces excessive ROS and may also favor the utilization of the peroxisomes and cytochromes for FAO. This ultimately facilitates disease progression by inducing oxidative stress and inflammation.

## Lipid export

In addition to FAO, export of triglycerides is the only way to reduce hepatic lipid content [68]. Due to their hydrophobic nature, fatty acids can only be exported from the liver after being packed into water-soluble VLDL particles alongside cholesterol, phospholipids, and apolipoproteins [110]. VLDL particles are formed in the endoplasmic reticulum, where apolipoprotein B100 (apoB100) is lipidated in a process catalyzed by the enzyme microsomal triglyceride transfer protein (MTTP). The nascent VLDL particle is then transferred to the Golgi apparatus, and during this process, the particle is further lipidated until a mature VLDL particle is formed [111]. While one molecule of apoB100 is associated with each VLDL particle, and is required for VLDL export, the triglyceride content of the VLDL particle can vary considerably [9, 110]. Consequently, apoB100 and MTTP are key components in hepatic VLDL secretion and in maintaining hepatic lipid homeostasis. As such, hepatic steatosis, secondary to compromised triglyceride export, is common in patients with genetic defects in the apoB or MTTP gene (hypobetalipoproteinemia and abetaproteinemia, respectively) [112, 113]. Although moderate exposure to fatty acids increased apoB100 secretion, prolonged exposure leads to ER stress and posttranslational degradation of apoB100, and consequently decreased apoB100 secretion, both in vivo and in vitro hereby linking ER stress to NAFLD progression through apoB100 inhibition [114, 115] (Fig. 3). MTTP gene transcription is positively regulated by PPARα and increased MTTP expression is paralleled by a change in apoB100 secretion, but a paradoxical decrease in circulating triglycerides in mice [116]. This could be due to a PPARα-mediated inhibition of apoCIII, promoting the clearance of apoB100-associated lipoproteins [116]. Notably, whereas PPARα agonism increases plasma HDL in humans, this is not the case in most applied rat and mouse models, as they lack the PPAR response element in the promotor region of ApoA1 (the major apolipoprotein of HDL); in fact, HDL may even be decreased in response to PPARα these species [117]. Thus, PPARα not only exerts its catabolic effect via FAO, but also through the regulation of lipoprotein metabolism. Conversely, both apoB100 and MTTP are negatively regulated by insulin, which reduces hepatic lipid export by inducing apoB100 degradation and suppressing MTTP synthesis [111]. High insulin levels in the post-prandial state decrease hepatic VLDL production, favoring chylomicron-mediated delivery of dietary lipids to the periphery [111], but the selective hepatic insulin resistance in patients with NAFLD allows insulin to stimulate DNL without inhibiting VLDL production [118]. VLDL secretion was increased in patients with NAFLD [65, 110, 119] and liver triglyceride content was directly associated with VLDL-TG secretion rates [65, 66, 110, 119]. However, while VLDL-TG export increased with intrahepatic lipid content, secretion plateaued when hepatic fat content exceeded 10%, surpassing the compensatory capacity to prevent increasing hepatic lipid accumulation [110]. Despite higher VLDL-TG secretion in patients with hepatic steatosis compared to healthy individuals, VLDL-apoB100 secretion was unchanged, suggesting that NAFLD patients do not secrete additional, but instead larger and more triglyceride-rich, VLDL particles [110]. However, very large VLDL particles cannot be secreted if their diameter exceeds that of the sinusoidal endothelial pores, and this limitation may ultimately result in lipid retention and NAFLD [120]. Failure to increase the number of secreted VLDL particles could indicate suboptimal apoB100 levels as a precipitating factor in NAFLD, and while mRNA levels of apoB100 and MTTP was found to be higher in patients with NAFLD compared to controls [35, 121], apoB100 synthesis rates were lower in patients with NASH (*n* = 7) compared to lean (*n* = 7) or BMI-matched obese (*n* = 7) controls without NASH [122]. Likewise, hepatic mRNA levels of apoB100 and MTTP and serum VLDL-TG were higher in patients with steatosis (*n* = 51) compared to patients with NASH (*n* = 53), marking deterioration of VLDL assembly and export as important in the progression from steatosis to NASH [75]. NAFLD patients with more advanced steatosis (> 30%) had decreased MTTP levels compared to healthy controls, suggesting that lipid export may also become compromised directly by the accumulation of substantial amounts of intracellular lipids [35]. Consequently, vector-mediated overexpression of MTTP in the Fatty Liver Shionogi mice decreased liver triglycerides and improved NASH [123]. Given the effect of PPARα on lipoprotein metabolism, it could be speculated that declining PPARα levels with NAFLD progression [75, 86] contribute to lower MTTP levels and apoB100 secretion rates. In contrast, similar expression levels of MTTP and apoB100 between patients with steatosis, NASH, and healthy controls have also been reported, denoting the considerable disease heterogeneity associated with NAFLD [124].

Despite the variation between studies, lipid export in NAFLD seems to be biphasic, initially increasing followed by a plateau or even decrease. The diminished export results in hepatic lipid overload and subsequent intracellular lipid accumulation, leading to steatosis, lipotoxicity, and liver damage, and promoting disease progression and fibrosis.

## Pre-clinical models and current clinical management

There is currently no approved pharmacological treatment for NAFLD. A major limiting factor in the development of new treatments is the lack of predictive pre-clinical models that accurately reflect human disease with regard to liver histology, pathophysiology, and metabolic abnormalities. The available animal models can be characterized as dietary, genetic or as a combination of these two. However, as mutations are rarely the cause of human NAFLD, this section will highlight some of the most commonly applied dietary models. These models attempt to replicate the unhealthy Western dietary pattern associated with NAFLD in humans, which are often high in fat, sugar, and cholesterol. However, to ensure translatability, it is important to maintain physiologically relevant levels of dietary macro- and micronutrients.

The methionine and choline-deficient diet (commonly applied in mice and rats) and choline-deficient L-amino-defined diet rapidly induce NASH, but fail to reproduce the pathophysiological response corresponding to clinical observations [125, 126]. Atherogenic diets can induce NASH and fibrosis, but the exceedingly high levels of cholesterol (1–2%) and the inclusion of cholic acid differ from the clinical situation and may even improve glucose tolerance and insulin sensitivity [127–129]. The addition of a high-fat component returns these to, at least, normal glucose/insulin levels [127]. Noticeably, a major limitation of the current models utilizing Western diets, not employing micronutrient deficiency or abnormally high amounts of cholesterol, is the absence of progressive, advanced hepatic fibrosis [129]. Using a human-like Western diet, the diet-induced animal model of non-alcoholic fatty liver disease (DIAMOND) mouse was developed as a promising pre-clinical model resembling human liver histology, pathophysiology, metabolic signatures, and advanced fibrosis as well as hepatocellular carcinoma after 52 weeks [130]. Unlike mice and rats, guinea pigs naturally resemble the human lipoprotein profile and develop human-like NASH histopathology, dyslipidemia, and hepatic oxidative stress when fed a Western diet [131–133]. Advanced hepatic fibrosis develops after 20–24 weeks [132], allowing interventions to be evaluated on advanced disease stages within a relatively short time frame. Nevertheless, while animals represent a tool to study NAFLD and NASH, it is unlikely that any single animal model will reflect all aspects of human NASH and researchers must critically select the model(s) best suited for the subject of investigation.

Diet and lifestyle interventions are mainstay in the treatment of NAFLD, and weight-loss ≥ 7% confers histological improvements of NASH [134]. However, lifestyle interventions are notoriously difficult to maintain [135], suggesting that some patients may benefit from pharmacological therapy. When validated in randomized clinical trials, only a few drugs have shown efficacy on NASH liver histology and/or fibrosis. These include vitamin E, pioglitazone (PPARγ agonist), obeticholic acid (farensoid X receptor agonist), cenicriviroc (CCR2/CCR5 antagonist), selonsertib (apoptosis signal-regulating kinase 1 inhibitor), and liraglutide (glucagon-like peptide 1 analogue) [136–141]. However, adverse effects may limit the treatment potential, e.g., glitazones are associated with phenotypical weight gain [136] and obeticholic acid induced pruritus and elevated LDL-C [137], the latter a potential concern in patients already at risk for cardiovascular disease. Cenicriviroc—despite not meeting its primary endpoint of NAFLD activity score improvement—[138] and obeticholic acid [137] were both able to improve hepatic fibrosis, and were currently undergoing phase III investigation. In addition, promising results have been reported for several phase II clinical trials and additional trials are currently ongoing, reflecting the growing research effort in developing novel treatments for NASH. Given the heterogenic nature of NAFLD, targeting metabolic, anti-inflammatory, or anti-fibrotic disease pathways simultaneously may exert an additive or synergistic effect and combination therapy may prove to be a an important tool in the future development of pharmacological treatment options.

## Conclusion

In NAFLD, hepatic lipid acquisition—mediated by increased fatty acid uptake and DNL—is enhanced despite the presence of steatosis. Lipid disposal may be increased, but is ultimately incapable of counteracting the growing intrahepatic fat deposition. While lipid export is enhanced in early disease stages, it decreases or plateaus with disease severity as hepatocyte metabolism becomes increasingly compromised. Efforts to reduce lipid levels can even promote disease progression, as FAO may induce oxidative stress, exhausting antioxidant competences and promoting damage to cellular organelles and DNA. The molecular mechanisms governing hepatic lipid homeostasis and the counter regulatory mechanisms related to a chronic lipid overload and NAFLD are both complex and tightly interconnected. Thus, any intervention targeting one or more pathway is likely to have consequences on multiple cellular signaling pathways. This, as well as inter-individual differences, should be taken into careful consideration when developing future treatment options for NAFLD.
